# Preclinical Identification Of Tumor-Draining Lymph Nodes Using a Multimodal Non-invasive *In vivo* Imaging Approach

**DOI:** 10.1007/s11307-022-01797-z

**Published:** 2023-01-04

**Authors:** Philipp Knopf, Dimitri Stowbur, Sabrina H. L. Hoffmann, Marieke F. Fransen, Johannes Schwenck, Bernd J. Pichler, Manfred Kneilling

**Affiliations:** 1 Werner Siemens Imaging Center, Department of Preclinical Imaging and Radiopharmacy, Eberhard Karls University, Tübingen, Germany; 2 Cluster of Excellence iFIT (EXC 2180) “Image Guided and Functionally Instructed Tumor Therapies”, 72076 Tübingen, Germany; 3 Department of Immunohematology and Blood Transfusion, Leiden University Medical Center (LUMC), Leiden, The Netherlands; 4 Department of Nuclear Medicine and Clinical Molecular Imaging, Eberhard Karls University, Tübingen, Germany; 5 German Cancer Consortium (DKTK) and German Cancer Research Center, Heidelberg, Germany; 6 Department of Dermatology, Eberhard Karls University, Tübingen, Germany

**Keywords:** Tumor-draining lymph nodes, PET/MRI, Optical imaging, ^18^F-FDG, Patent Blue V

## Abstract

**Purpose:**

Resection of the tumor-draining lymph -node (TDLN) represents a standard method to identify metastasis for several malignancies. Interestingly, recent preclinical studies indicate that TDLN resection diminishes the efficacy of immune checkpoint inhibitor-based cancer immunotherapies. Thus, accurate preclinical identification of TDLNs is pivotal to uncovering the underlying immunological mechanisms. Therefore, we validated preclinically, and clinically available non-invasive *in vivo* imaging approaches for precise TDLN identification.

**Procedures:**

For visualization of the lymphatic drainage into the TDLNs by non-invasive *in vivo* optical imaging, we injected the optical imaging contrast agents Patent Blue V (582.7 g mol^−1^) and IRDye® 800CW polyethylene glycol (PEG; 25,000–60,000 g mol^−1^), subcutaneously (s.c*.*) in close proximity to MC38 adenocarcinomas at the right flank of experimental mice. For determination of the lymphatic drainage and the glucose metabolism in TDLNs by non-invasive *in vivo* PET/magnetic resonance imaging (PET/MRI), we injected the positron emission tomography (PET) tracer (2-deoxy-2[^18^F]fluoro-D-glucose (^18^F-FDG) [181.1 g mol^−1^]) in a similar manner. For *ex vivo* cross-correlation, we isolated TDLNs and contralateral nontumor-draining lymph nodes (NTDLNs) and performed optical imaging, biodistribution, and autoradiography analysis.

**Results:**

The clinically well-established Patent Blue V was superior for intraoperative macroscopic identification of the TDLNs compared with IRDye® 800CW PEG but was not sensitive enough for non-invasive *in vivo* detection by optical imaging. *Ex vivo* Patent Blue V biodistribution analysis clearly identified the right accessory axillary and the proper axillary lymph node (LN) as TDLNs, whereas *ex vivo* IRDye® 800CW PEG completely failed. In contrast, functional non-invasive *in vivo* ^18^F-FDG PET/MRI identified a significantly elevated uptake exclusively within the ipsilateral accessory axillary TDLN of experimental mice and was able to differentiate between the accessory axillary and the proper LN. *Ex vivo* biodistribution and autoradiography confirmed our *in vivo* ^18^F-FDG PET/MRI results.

**Conclusions:**

When taken together, our results demonstrate the feasibility of ^18^F-FDG-PET/MRI as a valid method for non-invasive *in vivo*, intraoperative, and *ex vivo* identification of the lymphatic drainage and glucose metabolism within the TDLNs. In addition, using Patent Blue V provides additive value for the macroscopic localization of the lymphatic drainage both visually and by *ex vivo* optical imaging analysis. Thus, both methods are valuable, easy to implement, and cost-effective for preclinical identification of the TDLN.

**Supplementary Information:**

The online version contains supplementary material available at 10.1007/s11307-022-01797-z.

## Introduction

Identifying the correct tumor-draining lymph node (TDLN) is of paramount importance for malignant melanoma, breast carcinoma, head and neck, and prostate carcinoma patients [1–4]. Malignant melanoma patients with a vertical tumor thickness of ≥ 1.0 mm are designated for surgical resection of the TDLN [5–9]. Identification of macro- and micro-metastases within the draining LNs results in the resection of all LNs in the respective area and adjustment of the treatment regimen.

In this context, it has been shown that the identification of metastasis within TDLNs is associated with a poor prognosis. This standard of care treatment regimen was established long before novel and very efficient immune checkpoint inhibitor treatment protocols were implemented in the clinic. Therefore, we lack evidence concerning the impact of TDLN resection on immune checkpoint inhibitor treatment efficacy.

This information might be essential as Fransen MF. et al*.* and others have demonstrated, preclinically and clinically, that TDLNs are pivotal in immune checkpoint inhibitor treatment regimens [10, 11]. They revealed in an experimental adenocarcinoma model that therapeutic blockade of the PD-L1–PD-1 axis evoked dominant immune cell activation within the TDLN but not in the non-TDLN, resulting in homing, activation, and proliferation of CD8^+^ T cells in the tumor microenvironment of the adenocarcinomas. Interestingly, after surgical resection of the TDLN, the enhanced homing of CD8^+^ T cells into the adenocarcinomas was abrogated. In summary, resection of the TDLNs was associated with a loss of efficacy of an anti-PD-1/PD-L1 mAb treatment approach [11].

Consequently, it seems that TDLNs are of particular importance in maintaining and fostering a T-cell-driven antitumor immune response [12] to checkpoint-inhibitor therapies [11]. On the other hand, TDLNs and the lymphatic vascular network are heavily involved in lymphogenic tumor metastasis [13–15].

Thus, to gain deeper insights into the underlying molecular mechanisms within TDLNs, especially upon blockade of the PD-1–PD-L1 axis, a reproducible and easy non-invasive *in vivo* imaging method is required for precise preclinical identification of the TDLNs of interest.

Non-invasive ^99m^Tc-nanocolloid combined single-photon computed tomography and computed tomography (SPECT/CT), or TDLN scintigraphy, is the current clinical gold standard for identifying TDLNs [16]. After intradermal (i.d.) injection in close proximity to the tumor, ^99m^Tc-nanocolloid is transported via the lymphatic system into the TDLNs [17, 18]. After accumulation within the TDLN, ^99m^Tc-nanocolloid can be easily detected with SPECT/CT several hours before surgery and with a gamma probe during surgery.

Intratumoral (*i.t*.) or *i.d.* injection of Patent Blue V into the tumor margins represents another well-established clinical and FDA-approved method for intraoperative visual identification of the blue-stained TDLN [19–21].

However, the American Society of Breast Surgeons reported a 13% (95% CI: 9–18%, *I*^2^ = 36.7%) false-negative rate for the identification of the lymphatic stream TDLNs when using methylene blue, a similar agent, for the *in vivo* staining of TDLNs [22, 23]. Thus, in the clinic, the two methods, Patent Blue V and ^99m^Tc-nanocolloid, visualize the lymphatic stream and are routinely applied to tumor patients to avoid false-negative results and to improve the sensitivity concerning the identification of TDLNs. In the clinic, ^99m^Tc-nanocolloid is injected 1.5–3 h before SPECT/CT investigation [24, 25]. Patent Blue V is injected shortly before surgery [26]. In addition to these established methods, some investigators have evaluated the applicability of 2-deoxy-2[^18^F]fluoro-D-glucose (^18^F-FDG) for the identification of TDLNs, given that ^18^F-FDG is available in most centers and enables high-sensitivity positron emission tomography (PET)/CT or PET/magnetic resonance imaging (MRI). The main advantages of this approach include the superior sensitivity of PET and the additional anatomical information provided.

To our knowledge, we are the first to evaluate and compare different non-invasive *in vivo* multimodal imaging approaches, including two optical imaging agents (Patent Blue V and IRDye® 800CW polyethylene glycol (PEG)) and one PET tracer (^18^F-FDG), for the identification of TDLNs in a well-established tumor mouse model.

Thorek et al. have already demonstrated the potential of a multimodal nanoparticle, ^89^Zr-ferumoxytol, for the detection of LNs using PET/MRI [27]. Moreover, in another study, Thorek et al*.* demonstrated that i.d. ^18^F-FDG injection revealed lymphatic vessels and LNs using PET/CT. Combined with Cerenkov-guided intraoperative imaging, the accumulation of ^18^F-FDG provides sufficient Cerenkov radiation for the guidance of the resection of LN [28].

The compounds applied in our study differ heavily in their molecular weight (Patent Blue V (582.7 g mol^−1^), IRDye® 800CW PEG (PEG; 25,000–60,000 g mol^−1^), and ^18^F-FDG for PET (181.1 g mol^−1^)). In this context, it is important to consider that ^18^F-FDG represents a functional imaging method as it is taken up via specific glucose reporters by activated immune cells within the TDLNs, which exhibit a higher glucose metabolism [15, 29]. Furthermore, as determined in multiple preclinical studies, the TDLN is significantly enlarged with and without immune checkpoint inhibitor treatment and exhibits an enhanced glucose metabolism indicating immune cell activation [30, 31]. In contrast, both optical imaging compounds visualize the lymphatic drainage and thus nonspecifically accumulate within the TDLN.

Thus, our study aimed to evaluate and compare clinically available methods in a well-established small animal model of subcutaneous MC38 colon adenocarcinomas based on their ability to identify the TDLNs in the preclinical setting.

## Material and Methods

### Experimental Mice

C57BL/6 J mice were purchased from Charles River Laboratories (Sulzfeld, Germany) or bred at the animal facility of the medical faculty of the Eberhard Karls University Tübingen. Animals were housed in type 2 long individually ventilated cages (IVC) in standardized low-germ conditions with food (rodent pellet food) and water *ad libitum*. A maximum of 5 experimental mice were maintained in one IVC. Animals were provided with enrichment, such as timber houses and cellulose. In our *in vivo* studies, we used 8- to 10-week-old female and male mice.

### Experimental Exogenous Tumor Model

Tumors were inoculated on the right flank by subcutaneous (s.c.) injection of 0.5 × 10^6^ MC38 adenocarcinoma cells in PBS. Tumor volume was calculated on Day 7 (in mm^2^) by determination of the length and width using a caliper (Fine Science Tools GmbH, Heidelberg, Germany) and the following formula: $\mathrm{Tumor volume}=\frac{\mathrm{length}*{\mathrm{width}}^{2}}{2}$.

### Patent Blue V Phantom Measurements

Prior to Patent Blue V fluorescence *in vivo* and *ex vivo* optical imaging, we performed phantom studies to determine optimal filter settings using the IVIS® optical imaging system. We used 2 Patent Blue V phantoms: (1) 15 ml phantom (15 ml Falcon tube, Greiner Bio-One, Austria) with 4 µmol l^−1^ Patent V Blue in PBS with BSA; (2) 15 ml phantom with 4 µmol l^−1^ Patent V Blue in PBS without BSA. We used excitation filters (570 nm, 605 nm, 640 nm, 675 nm, 710 nm) and emission filters (620 nm, 640 nm, 680 nm, 700 nm, 720 nm, 740 nm, 760 nm, 780 nm) with medium binning, FOV: 25 cm and F-stop: 2 and different exposure times. The optimal excitation and emission filter setting (low exposure, low auto-fluorescence, and high excitation wave length) was used for further *in vivo* and *ex vivo* studies.

### In vivo/Ex vivo Optical Imaging Studies

For semiquantitative non-invasive *in vivo* identification of the TDLNs, we used a dedicated preclinical optical imaging system (*In Vivo* Imaging System (IVIS®) Spectrum optical imaging system, Perkin Elmer, Waltham, USA). In our studies, we applied two different optical imaging probes:

1. Patent Blue V (Guerbet, Roissy CdG Cedex, France; molecular weight: 582.7 g mol^−1^ [1]). Seven days after MC38 tumor cell inoculation, mice were injected s.c. in proximity to the MC38 tumor using 12.5 µl Patent V (25 mg ml^−1^). Five min after s.c. administration, fluorescence optical imaging was conducted using an exposure time of 1 s, an excitation wavelength of 675 nm, and an emission wavelength of 720 nm.
2. Analogously 25 µl IRDye® 800CW polyethylene glycol (PEG) (LI-COR Bioscience Nebraska, USA; molecular weight: PEG; 25,000–60,000 g mol^−1^), a near-infrared fluorescence imaging probe, diluted in PBS (0.1 nmol) was injected as described previously [13, 32, 33]. The manufacturer of IRDye® 800CW PEG has proven the visibility of surface blood vessels 30 min after *i.v.* administration of the contrast agent. As it is known that the large molecular weights of compounds such as IRDye® 800CW PEG (25,000–60,000 g mol^−1^) negatively affect clearance/tissue distribution, we used an IRDye® 800CW PEG drainage time of 30 min and only 5 min for Patent Blue V. Moreover, a pharmacokinetics study on IRDye® 800CW PEG was previously conducted by Bernhard et al. [34]. IRDye® 800CW PEG fluorescence optical imaging was performed using auto exposure, medium binning, an excitation wavelength of 745 nm, an emission wavelength of 800 nm, and a FOV of 14 cm. In total, twenty-five segments were utilized, including a delay of 1 min. *Ex vivo* optical imaging of the LNs of interest was performed using the abovementioned respective settings of the two optical imaging fluorescence probes.
  Auto exposure was chosen to obtain maximum photon exploitation. This value is normalized by calculation of the radiance (p/sec/cm2/sr). The obtained photons are calculated per second per square centimeter per steradian, allowing the normalization of the value and thus the comparison between Patent Blue V and IRDye 800 CW, despite using different exposure times. For non-invasive *in vivo* fluorescence optical imaging, mice were anesthetized with 1.5% isoflurane (CP Pharma, Burgdorf, Germany) with 100% oxygen within the IVIS® Spectrum optical imaging system with integrated temperature control to maintain the body temperature of the experimental mice. Imaging analysis was performed using Living Image Software 4.

### In vivo/Ex vivo PET/MRI Studies

For three-dimensional quantitative non-invasive *in vivo* visualization of the TDLNs [6, 7] by functional ^18^F-FDG-PET (^18^F-FDG: 181.1 g mol^−1^), we used a dedicated small animal microPET scanner (Siemens Heathineers, Knoxville, TN).

For non-invasive *in vivo* investigations, experimental mice underwent 1.5% isoflurane anesthesia (100% oxygen) in a specialized chamber with integrated temperature control 5–10 min prior to administration of ^18^F-FDG. 3.3–4.0 MBq ^18^F-FDG-PET was injected s.c. in proximity to the MC38 tumor 7 days after MC38 tumor cell inoculation. Sixty-minute dynamic PET scans were acquired 2 min after s.c. ^18^F-FDG injection (13 frames and an image size of 128 cm^2^). In addition, morphological coregistration was achieved to gain essential anatomical information enabling morphologic identification of the TDLNs using a small animal 7 T MRI system (Bruker) applying a 3D T2-TurboRARE sequence (matrix 256 × 256 × 64, TE 4.763 ms, TR 1800 ms, FOV 76.8 × 34.8 × 22.8 mm^3^, resolution 0.3 × 0.3 × 0.3 µm^3^, total acquisition time 9 min 7 s 200 ms). OSEM 3D was used for the reconstruction of the dynamic PET images. For a graphical representation of ^18^F-FDG uptake within the TDLNs, we selected the last frame between 50 and 60 min. A Gaussian filter with Inveon Research Workplace (Siemens Healthineers) was used for postprocessing and smoothing of the respective ^18^F-FDG-PET images. Additionally, we conducted *ex vivo* biodistribution analysis (γ-counting) with a general focus on the TDLNs and LNs. Here, ^18^F-FDG-related γ-counts were decay corrected and depicted as % injected dose (ID). For cross-correlation, autoradiography of the respective TDLN and NTDLN was performed.

### Statistical Analysis

GraphPad Prism software (Version 7.03, GraphPad Software, Inc., USA) was used for statistical analysis and graph generation. The mean value was calculated, and the standard error of the mean (SEM) was reported. The total number indicating the sample size (*n*) is demonstrated in the respective figure legend. Determination of statistical significance was performed using Tukey’s multiple comparison test with a confidence level of 95%. For significance level determination, commonly used notations (** corresponds to 0.001 ≤ *P* value < 0.01, * corresponds to 0.01 ≤ *P* value < 0.05, ns corresponds to a *P* value ≤ 0.05) were applied.

## Results

The precise identification of TDLNs is essential for continued studies aiming to uncover the precise role and underlying mechanisms of TDLNs in the context of immune checkpoint inhibitor therapy.

Thus, we aimed to identify the TDLNs of subcutaneous MC38 colon adenocarcinoma on the right flank of experimental mice by applying two different imaging modalities and three different compounds differing in their molecular weight and characteristics. Namely, Patent Blue V (582.7 g mol^−1^) and IRDye® 800CW polyethylene glycol (PEG; 25,000–60,000 g mol^−1^) were used for semiquantitative non-invasive *in vivo* optical imaging, and ^18^F-FDG (181.1 g mol^−1^) was used for quantitative non-invasive *in vivo* PET imaging. ^18^F-FDG-PET imaging reflects functional imaging (partly taken up by activated immune cells with elevated glucose metabolism [29, 35]) as well as visualization of the lymphatic vessels, lymph stream, and, consequently, TDLNs [29, 36]. Patent Blue V, as well as IRDye® 800CW PEG, labels the lymph stream and the TDLNs (Table 1).

**Table 1:** Imaging compounds for PET/MRI and optical imaging

Agent	Molecular weight	Modality	Reference
Patent Blue V	582.7 g mol^−1^ [68]	OI	[40]
IRDye® 800CW PEG	25,000–60,000 g mol^−1^ [69]	OI	[13, 70, 71]
^18^F-FDG	181.1 g mol^−1^ [72]	PET	[27, 49]

Three different compounds were evaluated concerning the feasibility for precise non-invasive *in vivo* and *ex vivo* identification of the TDLN of a s.c. MC38 colon adenocarcinoma at the right flank of an experimental mouse. ^18^F-FDG for PET imaging and Patent Blue V as well as IRDye® 800CW polyethylene glycol for fluorescence optical imaging

^18^F-FDG and Patent Blue V are FDA- and EMA-approved and clinically well-established compounds. ^18^F-FDG is mainly used for the identification of tumors and tumor metastasis, and Patent Blue V is used for the identification of TDLNs (Table 1). In addition, we examined the blood glucose levels before the injection of ^18^F-FDG injection, which ranged from 126 to 161 mg dl^−1^.

### Patent Blue V for Macroscopic Ex vivo Identification of TDLNs

**Fig. 1.**
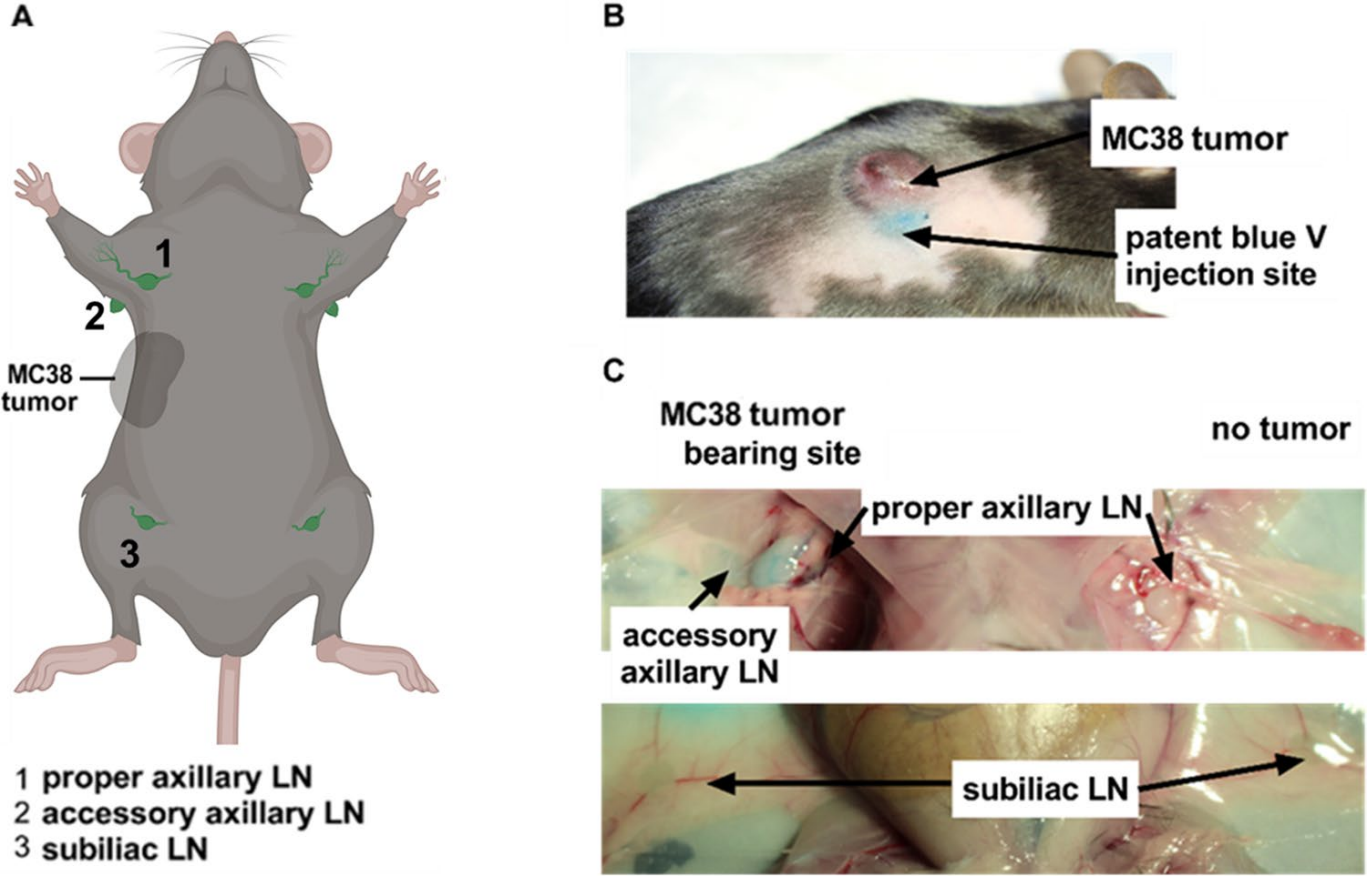
Localization of LNs in mice. **A** Schematic representation of the localization and nomenclature of murine LNs. **B** Exemplary image of a s.c. MC38 adenocarcinoma on the right flank of an experimental C57BL/6 J mouse s.c. injected with Patent Blue V in close proximity to the tumor. **C** *In situ* indication of the tumor-draining and nontumor-draining lymph nodes (TDLN and NTDLN, respectively) of an MC38 colon adenocarcinoma at the right flank, namely proper axillary LN, accessory axillary LN, and subiliac LN.

First, we applied Patent Blue V, a clinically validated dye that is frequently used for intraoperative labeling and identification of TDLNs after i.t., subdermal, or intracutaneous (i.c.) injection in breast and cervical carcinomas and malignant melanomas [37–39]. After a tumor volume of approximately 80 mm^3^ was achieved, we injected 12.5 µl Patent Blue V (25 mg ml^−1^) subcutaneously in the proximity of the s.c. MC38 adenocarcinoma at the right flank of experimental mice and sacrificed experimental mice immediately after the imaging procedure to identify the TDLNs *in situ*. In our experimental setup, we focused on three potential TDLNs located in proximity to the MC38 adenocarcinoma at the right flank of experimental mice, namely the ipsilateral (a) proper axillary, (b) accessory axillary, and (c) subiliac LNs, as depicted in Fig. 1.

As macroscopically illustrated in the representative image (Fig. 1C), Patent Blue V labeled the ipsilateral accessory axillary LN and the proper axillary LN as the main TDLN, given that the most pronounced Patent Blue V accumulation was noted within this LN. In this context, it is important to mention that the accessory axillary LN was notably enlarged compared to the proper axillary LN. Moreover, the accessory axillary LN is most probably located closer to the Patent Blue V injection site when compared to the proper axillary LN. A slight Patent Blue V accumulation was observed within the ipsilateral subiliac LN. In sharp contrast, no Patent Blue V accumulation was observed in the contralateral accessory axillary, proper axillary, and subiliac nontumor draining LNs (NTDLNs; Fig. 1C). Interestingly, Patent Blue V accumulation in the subiliac TDLN varied depending on the precise position of the MC38 adenocarcinoma and, thus, the Patent Blue V injection site.

### Patent Blue V Fluorescence Optical Imaging for Non-invasive In vivo and Ex vivo Identification of TDLNs

Next, we aimed to establish Patent Blue V fluorescence optical imaging for non-invasive *in vivo* identification of TDLNs. Thus, we first focused on phantom studies to establish an ideal workflow, including the optimal excitation and emission filter settings. Given that Patent Blue V emits light upon excitation exclusively when it is bound to serum albumin [40, 41], we used 15 ml phantom with 4 µmol l^−1^ Patent Blue V in PBS with 33 g l^−1^ BSA and 15 ml phantom with 4 µmol l^−1^ Patent Blue V in PBS (without BSA; negative control) in our phantom studies. These two phantoms underwent fluorescent optical imaging using different excitation and emission filter pairs, as shown in Figure S1. In experiments, we documented the exposure time for each filter pair (Figure S1A , B).

Thus, we selected a 675-nm excitation filter and a 720-nm emission filter for our intended non-invasive *in vivo* fluorescence optical imaging measurements due to the low exposure time of 3 s, the relatively high excitation wavelength, and the low autofluorescence signal.

**Fig. 2.**
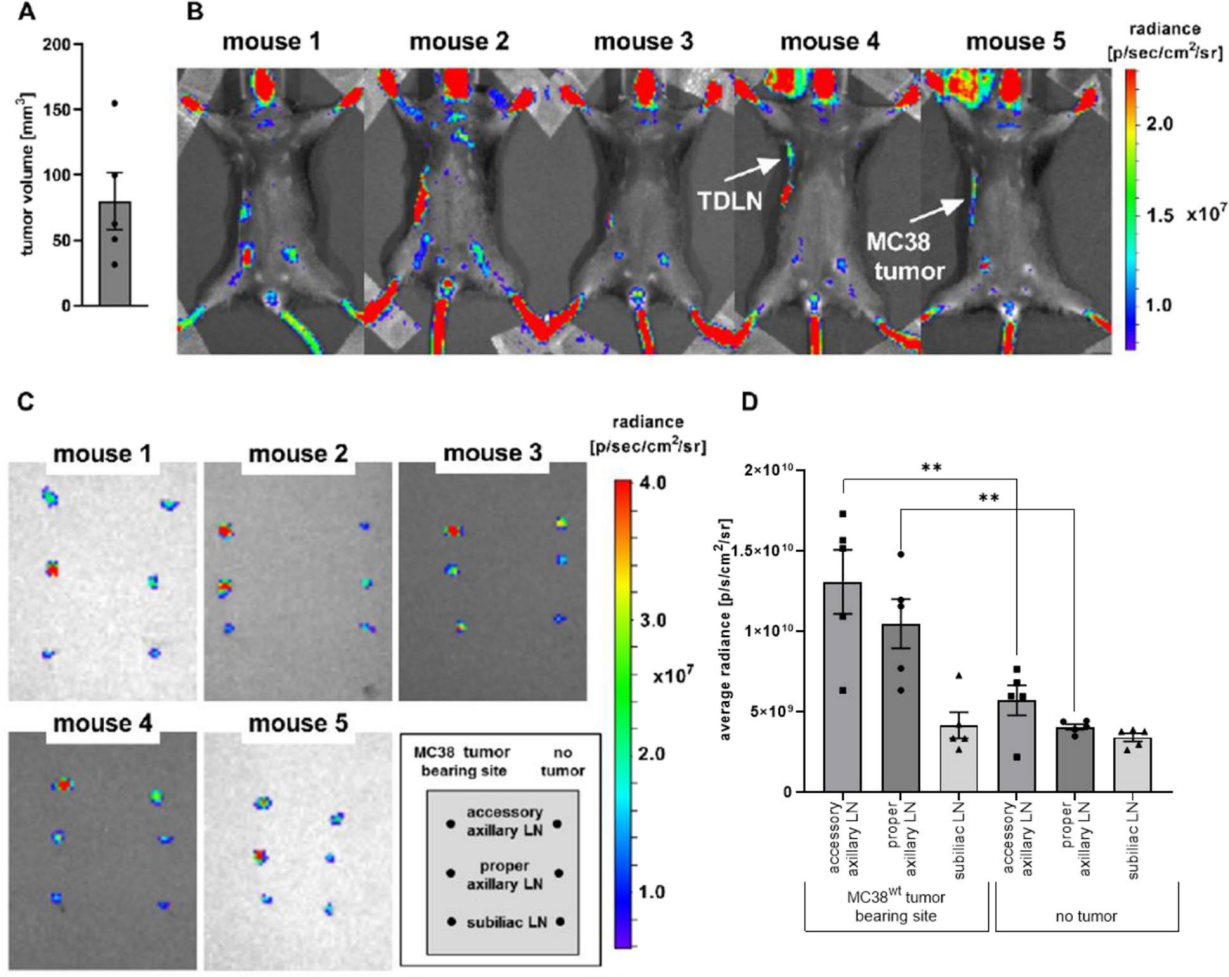
*In vivo* and *ex vivo* Patent Blue V OI of TDLNs. **A** MC38 tumor volumes (mm^3^) at Day 7 post cancer cell inoculation. **B** Mice bearing MC38 colon adenocarcinoma on the right flank were s.c. injected with Patent Blue V (25 mg ml^−1^) near the tumor for *in vivo* OI (ventral position, uptake time: 5 min, medium binning, FOV: 14, f-stop: 2, exposure: 10 s, excitation filter: 675 nm, emission filter: 720 nm). The tumor-draining lymph node (TDLN) and the MC38 tumor are indicated with an arrow. **C** Mice were sacrificed, and the indicated LNs were isolated and analyzed by *ex vivo* OI. D: *Ex vivo* quantification of Patent Blue V uptake in the LNs (n = 5 animals). Data are presented as the mean ± SEM. Statistics: Tukey’s multiple comparison test. LN = lymph node, TDLN = tumor-draining lymph node.

Next, we used our selected Patent Blue V fluorescence optical imaging settings for non-invasive *in vivo* investigations in MC38 adenocarcinoma-bearing C57BL/6 J mice with a tumor volume of approximately 80 mm^3^ (Fig. 2A). Five anesthetized mice were injected with Patent Blue V in proximity to the MC38 adenocarcinomas on the right flank, and non-invasive *in vivo* optical imaging was performed. One out of five animals demonstrated a slight fluorescence signal in the axilla of the ipsilateral site (Fig. 2B). Figure 2B exhibits the strong signal intensity observed in the neck, tail, and paws (the regions without black hair) of experimental mice, clearly indicating the systemic distribution of Patent Blue V within the body. In total, the *in vivo* fluorescence signal intensity was relatively weak and was probably affected by the limited penetration depth of approximately 4 mm, light absorption, and autofluorescence [14].

For *ex vivo* cross-correlation of the *in vivo* fluorescence signal intensity in experimental mice, the ipsilateral and contralateral accessory axillary, proper axillary, and subiliac TDLNs were dissected for *ex vivo* optical imaging analysis using the previously implemented optical imaging settings (Fig. 2C).

The ipsilateral accessory axillary and the proper axillary TDLN exhibited enhanced Patent Blue V accumulation compared to the NTDLNs of the contralateral site (Fig. 2C). The *ex vivo* quantification of the fluorescence signal intensity in the respective LNs revealed a significantly increased signal intensity in the ipsilateral accessory axillary and proper axillary LNs compared to their contralateral NTDLN counterparts. Despite marginal Patent Blue V accumulation in the ipsilateral subiliac TDLNs, no significant difference in Patent Blue V signal intensity between the ipsilateral subiliac TDLNs and the contralateral NTDLNs was observed (Fig. 2C, D).

**Fig. 3:**
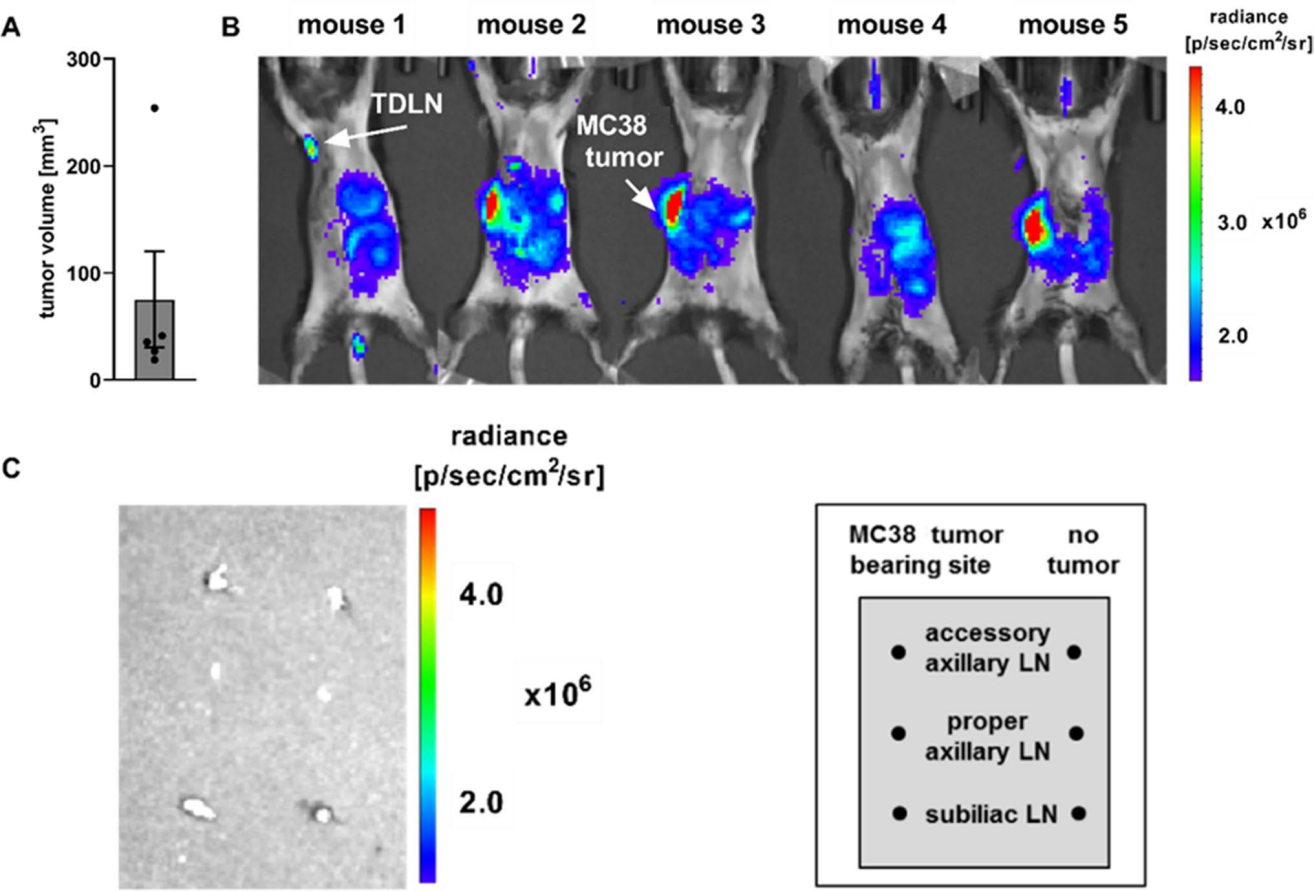
*In vivo* IRDye® 800CW PEG OI of TDLNs. **A** MC38 tumor volumes (mm^3^) at day 7 post cancer cell inoculation. **B** Mice bearing MC38 colon adenocarcinoma at the right flank were s.c. injected with IRDye® 800CW PEG (~ 0.1 nmol; [44]) near the tumor. *In vivo*, OI was performed 30 min post-IRDye® 800CW PEG injection (ventral position, *n* = 5 animals, medium binning, FOV: 25, f-stop: 2, auto exposure, excitation filter: 745 nm, emission filter: 800 nm). The tumor-draining lymph node (TDLN) and the MC38 tumor are indicated with an arrow. **C** Mice were sacrificed, and the indicated LNs were isolated and analyzed by *ex vivo* OI. Data are presented as the mean ± SEM. Statistics: Tukey’s multiple comparison test. LN = lymph node, TDLN = tumor-draining lymph node.

Next, the NIR contrast agent IRDye® 800CW conjugated to PEG was studied. The molecular weight of IRDye® 800CW PEG ranges between PEG; 25,000–60,000 g mol^−1^ and is therefore markedly larger than the molecular weight of the Patent Blue V sodium salt [42, 43]. Similar to Patent Blue V, mice were injected with IRDye® 800CW PEG s.c. in proximity to MC38 tumors with an approximate size of 2.32 mm (diameter) on the right flank of the experimental mouse (Fig. 3A). *In vivo* imaging revealed an accumulation of IRDye® 800CW PEG in the axilla on the tumor-bearing site of one out of five mice (Fig. 3B). Given that a strong signal intensity was detected at the injection site, the absence of IRDye® 800CW PEG uptake in the LNs most likely does not reflect a detection problem but maybe an insufficient or weaker accumulation of the dye in the TDLN compared to Patent Blue V. This finding could be related to the larger molecular weight of IRDye® 800CW PEG, which could impede or delay lymph vessel penetration. Although used as a contrast agent for *in vivo* visualization of LNs by others [13, 44], *ex vivo* analysis revealed no IRDye® 800CW PEG NIR signal either in the TDLN or in the NTDLN (Fig. 3C). In this regard, Proulx et al*.* reported a slow clearance of the dye from the injection site based on a higher molecular weight [13]. Therefore, an even longer uptake duration might be recommended for IRDye® 800CW PEG OI measurements.

When taken together, these results demonstrate that Patent Blue V OI was exclusively qualified to identify the accessory axillary and proper axillary LNs as the main TDLNs.

### Non-invasive In vivo Imaging of TDLNs with ^18^F-FDG-PET/MRI

Next, we evaluated the broadly available clinical routine for the staging of patients with malignancies using the well-established and inexpensive tracer ^18^F-FDG and combined PET/MRI. This methodology enables three-dimensional, quantitative, and high-resolution imaging. Thus, the feasibility of identifying the TDLNs of s.c. MC38 adenocarcinomas on the right flank of experimental mice using the previously described setup were assessed. Importantly, to our knowledge, there are no reports of metastasis (including the LNs) of MC38 adenocarcinoma cells. Therefore, the ^18^F-FDG uptake in TDLNs over the 60 min dynamic PET scan is not related to LN metastasis of the MC38 adenocarcinomas but rather to activated metabolic active immune cells.

The rationale for this study was to overcome the limitations of OI in terms of light absorption, penetration depth, and the lack of three-dimensional quantitative imaging by PET/MRI. Combined PET/MRI measurements enable functional ^18^F-FDG-PET imaging of activated immune cells with enhanced glucose metabolism within the TDLN together with MRI, which is well qualified to provide essential anatomical information about the exact anatomic site of ^18^F-FDG uptake. Thus, combined ^18^F-FDG-PET/MRI might represent a clinically highly relevant approach for identifying TDLNs.

**Fig. 4:**
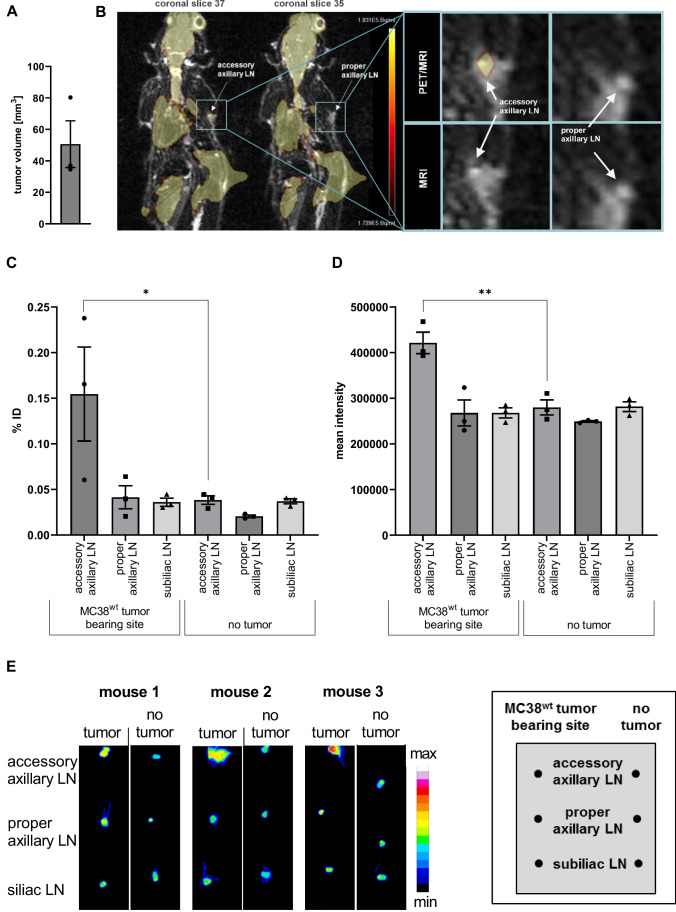
Imaging of TDLNs by ^18^F-FDG-PET/MRI *in vivo* and *ex vivo*. **A** MC38 tumor volumes (mm³) at day 7 post cancer cell injection. **B** Mice bearing an MC38 colon adenocarcinoma at the right flank were injected with ^18^F-FDG near the tumor for *in vivo* PET/MR imaging. Representative PET images of the last 10-min frame of a 60-min dynamic PET scan were merged with the respective MR image, indicating tumor-draining (accessory axillary LN, coronal slice 37) and nontumor-draining (proper axillary, coronal slice 35) LNs. **C** %ID corrected for radioactive decay of tumor-draining and nontumor-draining accessory axillary, proper axillary and subiliac LNs. **D** Quantitative *ex vivo* 18F-FDG-autoradiography analysis and **E** autoradiography images of the respective LNs (*n* = 3 animals). Data are presented as the mean ± SEM. Statistics: Tukey’s multiple comparison test. LN = lymph node, TDLN = tumor-draining lymph node, NTDLN = nontumor-draining lymph node.

In experiments, mice were injected s.c. with 3.3–4.0 MBq ^18^F-FDG in proximity to the s.c. MC38 tumors with an approximate volume of 50 mm^3^ at the right flank of experimental mice. Non-invasive *in vivo* ^18^F-FDG-PET/MRI revealed significantly higher ^18^F-FDG uptake in the ipsilateral axilla of the tumor-bearing experimental axilla than in the contralateral axilla (Fig. 4A, B). LNs were identified based on the MR images. Thus, we were able to differentiate the ^18^F-FDG uptake of the accessory axillary LNs from the proper axillary LNs, which would not have been possible without MRI. In this context, it is necessary to mention again that the accessory axillary LN is most probably located closer to the Patent Blue V injection site than the proper axillary LN. Moreover, no ^18^F-FDG uptake in the ipsilateral subiliac LN was detectable by PET/MRI. *Ex vivo* biodistribution analysis (γ-counting) of the ^18^F-FDG uptake within the LNs of interest identified the accessory axillary LN as the main TDLN of a s.c. MC38 adenocarcinoma at the right flank of experimental mice and consequently confirmed our findings taken by the non-invasive *in vivo* ^18^F-FDG PET/MRI. In addition, ^18^F-FDG autoradiography analysis of the LNs of interest confirmed our *ex vivo* biodistribution results of the respective TDLNs and NTDLNs and thus identified the accessory axillary LNs as TDLNs (Fig. 4C, E). Most importantly, the accessory axillary LN was strongly enlarged compared to the proper axillary LN as indicated in Fig. 4B and E. Moreover, an enlarged TDLN exhibits an enhanced immune cell activation state and, thus an enhanced glucose metabolism of the involved activated immune cells [30, 31].

Our data thus indicate that non-invasive *in vivo* ^18^F-FDG-PET/MRI represents a superior tool to easily identify metabolically active TDLNs of experimental mice.

## Discussion

With the emergence of immune checkpoint inhibitor therapies, which are based on the engagement of the host immune system to battle against cancer, the identification of primary as well as secondary lymphoid organs via non-invasive *in vivo* imaging has gained increasing importance in recent years [45]. In a translational study, Schwenck et al. were able to identify responders to immune checkpoint inhibitor-based cancer immunotherapy in tumor-bearing experimental mice and metastatic melanoma patients based on responder-specific metabolic changes in glucose metabolism within the bone marrow and spleen non-invasively *in vivo* determined by whole-body ^18^F-FDG-PET/CT imaging [45]. This essential role of the secondary lymphatic organs, such as the TDLNs, is further supported by a study from Fransen et al*.,* highlighting that immune cell homing and immune cell activation within the TDLN upon anti-PD-L1 mAb therapy is pivotal for an efficient treatment response [11]. Another recent publication by Wu et al. indicated that activated T cells, which are characterized by high glucose metabolism and lactic acid production, create acidic niches within LNs and thereby suppress T-cell effector functions associated with reduced IFN-γ or IL-2 cytokine release. T cell functioning is thus subject to immune regulation by acidification of paracortical zones within the LNs [46]. These studies accentuate the special importance of secondary lymphatic organs, such as TDLNs, and consequently the need to identify TDLNs by non-invasive in vivo imaging in a preclinical as well as clinical setting. In recent years, several preclinical and clinical studies have focused on labeling lymphatic vessels and thus TDLNs by injection of Isosulfan Blue, Patent Blue V, Evans Blue, technetium-99 m (^99m^Tc) or ^18^F-FDG in close proximity to the respective tumor [47–49]. The respective TDLNs were subsequently identified macroscopically by the eye or by optical imaging (Isosulfan Blue, Patent Blue V, Evans Blue), SPECT (^99m^Tc) or PET/CT as well as PET/MRI (^18^F-FDG) [47–50].

Thorek et al*.* successfully identified lymphatic vessels and LNs by ^18^F-FDG PET/Cerenkov imaging and demonstrated the potential of PET/Cerenkov-guided LN resection [28]. Cerenkov-guided TDLN identification would be a useful, promising, and innovative approach. Later, Lockau et al. successfully identified even LN metastases using *in vivo* ^18^F-FDG PET lymphography in B16-F10 melanoma-bearing mice [49]. Our study focused on the validation of the suitability and the comparison of the two fluorescent dyes, Patent Blue V and IRDye® 800CW PEG for OI as well as of ^18^F-FDG for combined PET/MRI to identify the TDLNs in experimental mice with a solid subcutaneous MC38 adenocarcinoma tumor located on the right flank.

The OI dyes Patent Blue V and IRDye® 800CW PEG were not qualified for sensitive and reliable *in vivo* identification of TDLNs, given that we were only able to visualize TDLNs in one out of five Patent Blue V-injected and in only one out of five IRDye® 800CW PEG-injected experimental mice.

Patent Blue V was suitable for reliable and sensitive *in situ* and *ex vivo* identification of the TDLNs, namely, the accessory and axillary LNs (Fig. 2). In sharp contrast, IRDye® 800CW PEG was not qualified for reliable and sensitive in situ and *ex vivo* identification of the TDLNs. One reason for the lack of IRDye® 800CW PEG accumulation in TDLNs might be the higher molecular weight compared to Patent Blue V (25,000–60,000 g mol^−1^ vs. 582.7 g mol^−1^ [44, 51]) but also other molecular characteristics such as hydrophobia or molecular charge can have an impact. Gretz et al. showed that molecules with a lower molecular weight enter the LN cortex and disperse through the reticular network due to improved tissue penetration, whereas molecules with a high molecular weight are unable to access the cortex [52, 53]. Contrary to our results, data from Krishnan et al. revealed that IRDye® 800CW PEG is qualified for the *in vivo* identification of lymphatic vessels and draining LNs as well as blood vessels when injected intravenously into patients with oral squamous cell carcinoma [54].

In the experimental setup of our study, the accumulation in the TDLNs might be significantly slower due to the high molecular weight of IRDye® 800CW PEG in combination with the s.c. administration route. As stated above, this finding is related to the observation that molecules with a high molecular weight distribute within the draining LN less strongly than molecules with a low molecular weight [52]. The clinically approved NIR dye indocyanine green with a molecular weight similar to that of Patent Blue V (775 g mol^−1^) [55] has been extensively and successfully applied for the *in vivo* identification of TDLNs in several animal species as well as in humans [56–59]. In contrast to IRDye® 800CW, which is conjugated to PEG, indocyanine green is an unconjugated fluorophore resulting in a lower molecular weight, which might lead to a fast and, therefore, more pronounced uptake of the contrast agent in the TDLN. Similar to other NIR dyes, indocyanine green is characterized by reduced background autofluorescence and increased tissue penetration [58], representing an advantage over Patent Blue V based on the emission of light in the visible light spectrum. Nevertheless, the OI of Patent Blue V was applicable to identify the TDLNs *in situ* and *ex vivo* in a fast and cost-effective manner. Taken together, Patent Blue V, IRDye® 800CW PEG, and indocyanine green accumulate via the lymphatic vessels within the TDLN and therefore do not provide functional information on the immune cell activation state or glucose metabolism.

In addition to the two evaluated OI dyes, we evaluated whether s.c. injection of ^18^F-FDG near the MC38 adenocarcinoma together with PET/MRI is a qualified and sensitive tool to identify the TDLNs. In contrast to Patent Blue V and IRDye® 800CW PEG, which indirectly label the lymph stream and thus the draining LN, the radiotracer ^18^F-FDG is taken up by cells with enhanced glucose demand and glucose metabolism, such as tumor cells, resident cells, and immune cells. LNs such as TDLNs are mainly composed of immune cells (T cells, B cells, etc.). ^18^F-FDG in the TDLNs (without LN metastasis) is taken up almost exclusively by the mentioned immune cells. In this regard, it is important to consider that activated T cells within the TDLN directed against tumor-associated antigens exhibit enhanced glucose metabolism and thus take up more ^18^F-FDG than LNs without activated T cells [60]. In our study, non-invasive *in vivo* PET/MRI 30 min after s.c. injection of ^18^F-FDG near the tumor at the right flank exhibited enhanced ^18^F-FDG uptake within the right axilla of the MC38 tumor-bearing mice. In contrast to Patent Blue V, where the accessory axillary and the proper axillary LN were identified as TDLNs by *ex vivo* OI, *ex vivo* ^18^F-FDG biodistribution and autoradiography analysis exclusively identified the accessory axillary LN as the main TDLN (Fig. 4C–E).

The difference between *in situ*/*ex vivo* Patent Blue V OI biodistribution analysis and *ex vivo* ^18^F-FDG biodistribution and autoradiography analysis might be due to the different characteristics of the two applied imaging agents, as ^18^F-FDG is taken up predominantly by immune cells with an increased metabolic glucose demand, suggesting that immune cell homing and activation rather occurs in the accessory axillary LN [61, 62]. In contrast to non-invasive *in vivo* OI, combined non-invasive *in vivo* ^18^F-FDG-PET/MRI allows the detection of functional TDLNs with high glucose metabolism and furthermore provides anatomical information on the exact location of the LN at high spatial resolution [47]. Compared to ^18^F-FDG-PET/MRI, non-invasive *in vivo* Patent Blue V OI was not applicable to differentiate between accessory axillary and proper axillary LNs and thus required *ex vivo* quantification. As the most common clinically applied PET tracer, ^18^F-FDG has also been applied by Singh et al. to identify sentinel LNs in patients with metastatic malignant melanoma. In this context, Singh et al. reported that non-invasive *i.v.* (anterior cubital vein) preoperative ^18^F-FDG-PET/CT imaging cannot serve as a substitute for lymphoscintigraphy with ^99m^Tc-nanocolloid (sensitivity: 100%) due to the low sensitivity of ^18^F-FDG-PET (sensitivity: 14.3%; 95% CI, 2.5 to 44%) [63], which might be associated with the low diameter of the metastatic nodules, as indicated by Crippa et al. In this patient cohort, only 23% of LN metastases with a diameter less than 5 mm were identified by ^18^F-FDG-PET. In contrast, 83% of LN metastases with a diameter of 6–10 mm and 100% of LN metastases with a diameter greater than 10 mm were identified by ^18^F-FDG-PET [64].

The German procedural instructions for the identification of TDLNs recommend the use of ^99m^Tc-nanocolloid, which should be injected i.c. in close proximity to the melanoma, followed by non-invasive SPECT/CT and *in situ* lymphoscintigraphy during surgery. A meta-analysis of seventeen comparative ^99m^Tc-nanocolloid-SPECT/CT and lymphoscintigraphy studies, including 1438 patients revealed a slightly higher sensitivity of SPECT/CT (98.28%) in comparison to lymphoscintigraphy (95.53%) [48]. Additionally, in most clinics melanoma patients will be i.c. injected near the tumor with Patent Blue V, as Patent Blue V is a highly recommended method for the identification of TDLNs with a high sensitivity (< 95%) and a low false-negative rate (5–10%) [65].

I.c. injection of ^99m^Tc-nanocolloid and Patent Blue V within one patient with malignant melanoma can reveal contradictory results. It is possible that Patent Blue V strongly accumulates and macroscopically dyes the TDLN, whereas no or only a low ^99m^Tc-nanocolloid concentration can be detected within the identical TDLN or vice versa. This observation is probably the consequence of the different molecular weights of both agents, as the molecular weight highly determines the grade of tissue distribution [53].

Based on German procedural instructions for nuclear medical sentinel LN diagnosis in patients suffering from melanoma, breast carcinoma, head, and neck cancer, or prostate carcinoma, the usage of ^99m^Tc-nanocolloid represents the primary indication for TDLN identification [1–4].

In addition to immunotherapies, there is great interest in identifying TDLNs for sentinel LN resection to anticipate metastatic spread or detect LN metastases. In this regard, Bae et al. claim that ^18^F-FDG PET/CT is superior to CT/MRI or CT or MRI alone in terms of LN metastasis identification in patients with oral cavity squamous cell carcinoma [66]. In addition to these clinical studies, ^18^F-FDG is nonspecifically taken up by cells with elevated metabolic demand, including T cells and tumor metastases in the LN.

Identification of the TDLNs might be of special importance for upcoming novel therapeutic approaches with a focus on intranodal administration of immune checkpoint inhibitors or oncolytic viruses to improve the therapy outcome of cancer patients [31, 67]. Furthermore, immunotherapy would foster immune cell activation and, therefore, enlarge the TDLN and thus enhance the ^18^F-FDG uptake.

In summary, Patent Blue V-OI and ^18^F-FDG-PET/MRI identified the accessory axillary LN on the ipsilateral site of the tumor-bearing mice as the main TDLN.

For extensive continuative preclinical studies with a focus on the role of the TDLN during immune checkpoint inhibitor therapy, we recommend an initial cheap and easy investigation by injecting Patent Blue V s.c. at the respective areas near the tumor to enable *in situ* and *ex vivo* identification of the lymphatic drainage and, consequently, the TDLN. Secondly, we recommend functional three-dimensional quantitative high-resolution ^18^F-FDG PET/MRI investigations to reveal the drainage of ^18^F-FDG into the TDLN along with the associated glucose metabolism.

Combining Patent Blue V OI and ^18^F-FDG-PET/MRI would be very helpful in the preclinical setting. Thus, we recommend the first s.c. ^18^F-FDG-injections in proximity to the tumor followed by immediate (2 min.) PET/MRI over 30–60 min. Afterward, Patent Blue V should be s.c. injected into the experimental mice at the identical site of the ^18^F-FDG-injection. 5 min later, mice should be sacrificed and dissected and LNs of interest semi-quantitatively analyzed by OI and by γ-counting. These investigations could be linked to continuative flow cytometry analysis of the immune cell population of the TDLNs and NTDLNs.

Patent Blue V OI and visual identification of the TDLN and metabolic identification of the TDLN by ^18^F-FDG might provide two different yet essential pieces of information. Therefore, continuative studies are required to differentiate the role of the Patent Blue V accumulating TDLNs and the exclusively ^18^F-FDG accumulating TDLN (accessory axillary LN) from the role of the ^18^F-FDG non-accumulating but Patent Blue V accumulating TDLN (proper axillary LN) in regard of the efficacy of an immune checkpoint inhibitor-based cancer.

Our results demonstrate the feasibility of ^18^F-FDG-PET/MRI as a valid method for non-invasive *in vivo* and *ex vivo* identification of TDLNs. However, the additional use of Patent Blue V provides an additive value for macroscopic identification of the TDLN by the eye or by *ex vivo* optical imaging analysis. Both methods are valuable, easy to implement, and cost-effective. These findings might have broad preclinical and clinical implications for LN resections and the consequences for immune checkpoint inhibitor therapies, as reported by Fransen et al*.* [11].

## Supplementary Information

Below is the link to the electronic supplementary material.

Supplementary file1 (DOCX 410 KB)

## Data Availability

Data from Fransen et al. advocates the importance of the TDLN identification to optimally engage the antitumor immune response (by checkpoint inhibitor therapies) and thereby enhance clinical benefit. Data availibility regarding this reference is confirmed by the author.

## References

[1] Buscombe J (2007). Sentinel node in breast cancer procedural guidelines. Eur J Nucl Med Mol Imaging.

[2] Wengenmair H et al (2002) [Sentinel lymph node diagnosis in prostatic carcinoma: II. Biokinetics and dosimetry of 99mTc-Nanocolloid after intraprostatic injection]. Nuklearmedizin 41(2):102–7 11989296

[3] Bachter D (2001). The predictive value of the sentinel lymph node in malignant melanomas. Recent Results Cancer Res.

[4] Koch WM (1998). Gamma probe-directed biopsy of the sentinel node in oral squamous cell carcinoma. Arch Otolaryngol Head Neck Surg.

[5] Chatterjee A, Serniak N, Czerniecki BJ (2015). Sentinel lymph node biopsy in breast cancer: a work in progress. Cancer J.

[6] Narayanan R, Wilson TG (2018). Sentinel node evaluation in prostate cancer. Clin Exp Metastasis.

[7] Höft S (2004). Sentinel lymph-node biopsy in head and neck cancer. Br J Cancer.

[8] Meads C (2014). Sentinel lymph node biopsy in vulval cancer: systematic review and meta-analysis. Br J Cancer.

[9] Thomas JM (2008). Sentinel lymph node biopsy in malignant melanoma. BMJ.

[10] Dammeijer F (2020). The PD-1/PD-L1-Checkpoint restrains T cell immunity in tumor-draining lymph nodes. Cancer Cell.

[11] Fransen MF (2018). Tumor-draining lymph nodes are pivotal in PD-1/PD-L1 checkpoint therapy. JCI insight.

[12] Rotman J (2019). Unlocking the therapeutic potential of primary tumor-draining lymph nodes. Cancer Immunol Immunother.

[13] Proulx ST (2013). Use of a PEG-conjugated bright near-infrared dye for functional imaging of rerouting of tumor lymphatic drainage after sentinel lymph node metastasis. Biomaterials.

[14] Hoshida T (2006). Imaging steps of lymphatic metastasis reveals that vascular endothelial growth factor-c increases metastasis by increasing delivery of cancer cells to lymph nodes: therapeutic implications. Can Res.

[15] Gillot L (2021). The pre-metastatic niche in lymph nodes: formation and characteristics. Cell Mol Life Sci.

[16] Israel O (2019). Two decades of SPECT/CT – the coming of age of a technology: An updated review of literature evidence. Eur J Nucl Med Mol Imaging.

[17] Valdés Olmos RA, Rietbergen DDD, Vidal-Sicart S (2014). SPECT/CT and sentinel node lymphoscintigraphy. Clin Transl Imaging.

[18] Brouwer OR (2012). Comparing the hybrid fluorescent–radioactive tracer indocyanine green–<sup>99m</sup>Tc-nanocolloid with <sup>99m</sup>Tc-nanocolloid for sentinel node identification: a validation study using lymphoscintigraphy and SPECT/CT. J Nucl Med.

[19] Pelizzo MR (2001). The sentinel node procedure with Patent Blue V dye in the surgical treatment of papillary thyroid carcinoma. Acta Otolaryngol.

[20] Ansink AC et al (1999) Identification of sentinel lymph nodes in vulvar carcinoma patients with the aid of a patent blue V injection: a multicenter study. Cancer 86(4):652–656 10.1002/(sici)1097-0142(19990815)86:4<652::aid-cncr14>3.0.co;2-r 10440693

[21] Yap YL (2009). Patent blue dye in lymphaticovenular anastomosis. Ann Acad Med Singap.

[22] Schmid P (2018). Atezolizumab and nab-paclitaxel in advanced triple-negative breast cancer. N Engl J Med.

[23] Simmons R (2003). Methylene blue dye as an alternative to isosulfan blue dye for sentinel lymph node localization. Ann Surg Oncol.

[24] Seo Y (2011). Mapping of lymphatic drainage from the prostate using filtered <sup>99m</sup>Tc-sulfur nanocolloid and SPECT/CT. J Nucl Med.

[25] KleinJan GH (2018). The best of both worlds: a hybrid approach for optimal pre- and intraoperative identification of sentinel lymph nodes. Eur J Nucl Med Mol Imaging.

[26] Brouwer OR (2012). Comparing the hybrid fluorescent-radioactive tracer indocyanine green-99mTc-nanocolloid with 99mTc-nanocolloid for sentinel node identification: a validation study using lymphoscintigraphy and SPECT/CT. J Nucl Med.

[27] Thorek DL (2014). Non-invasive mapping of deep-tissue lymph nodes in live animals using a multimodal PET/MRI nanoparticle. Nat Commun.

[28] Thorek DL (2012). Positron lymphography: multimodal, high-resolution, dynamic mapping and resection of lymph nodes after intradermal injection of 18F-FDG. J Nucl Med.

[29] Pektor S (2021). Characterization of activation induced [18]F-FDG uptake in Dendritic Cells. Nuklearmedizin.

[30] MartIn-Fontecha A (2003). Regulation of dendritic cell migration to the draining lymph node: impact on T lymphocyte traffic and priming. J Exp Med.

[31] Fransen MF, van Hall T, Ossendorp F (2021) Immune checkpoint therapy: tumor draining lymph nodes in the spotlights. Int J Mol Sci 22(17):9401 10.3390/ijms22179401 PMC8431673 34502307

[32] Weiler M, Dixon JB (2013) Differential transport function of lymphatic vessels in the rat tail model and the long-term effects of Indocyanine Green as assessed with near-infrared imaging. Front Physiol 4:215 10.3389/fphys.2013.00215 PMC3744037 23966950

[33] Carr JA (2018). Shortwave infrared fluorescence imaging with the clinically approved near-infrared dye indocyanine green. Proc Natl Acad Sci U S A.

[34] Bernhard W (2021). Preclinical study of IRDye800CW-nimotuzumab formulation, stability, pharmacokinetics, and safety. BMC Cancer.

[35] Chang MC (2012). 18F-FDG PET or PET/CT for detection of metastatic lymph nodes in patients with endometrial cancer: a systematic review and meta-analysis. Eur J Radiol.

[36] Van den Broeck W, Derore A, Simoens P (2006). Anatomy and nomenclature of murine lymph nodes: Descriptive study and nomenclatory standardization in BALB/cAnNCrl mice. J Immunol Methods.

[37] Kieckbusch H (2008). Patent blue sentinel node mapping in cervical cancer patients may lead to decreased pulse oximeter readings and positive methaemoglobin results. Eur J Anaesthesiol.

[38] Johnson S, Arora S, Babu E (2012). Injecting patent blue dye V for sentinel lymph node biopsy without skin staining. Ann R Coll Surg Engl.

[39] Gallegos-Hernández JF (1998). Identification of sentinel lymph node with patent blue V in patients with cutaneous melanoma. Gac Med Mex.

[40] Tellier F (2012). Sentinel lymph nodes fluorescence detection and imaging using Patent Blue V bound to human serum albumin. Biomed Opt Express.

[41] Le CP (2016). Chronic stress in mice remodels lymph vasculature to promote tumour cell dissemination. Nat Commun.

[42] Danhier P (2015). Combining optical reporter proteins with different half-lives to detect temporal evolution of hypoxia and reoxygenation in tumors. Neoplasia.

[43] Ntziachristos V (2000). Concurrent MRI and diffuse optical tomography of breast after indocyanine green enhancement. Proc Natl Acad Sci.

[44] Huntington JA, Stein PE (2001). Structure and properties of ovalbumin. J Chromatogr B Biomed Sci Appl.

[45] Schwenck J (2020). Cancer immunotherapy is accompanied by distinct metabolic patterns in primary and secondary lymphoid organs observed by non-invasive in vivo (18)F-FDG-PET. Theranostics.

[46] Wu H (2020). T-cells produce acidic niches in lymph nodes to suppress their own effector functions. Nat Commun.

[47] Zhang F (2011). Preclinical lymphatic imaging. Mol Imaging Biol.

[48] Quartuccio N et al (2020) Comparison of (99m)Tc-labeled colloid SPECT/CT and Planar lymphoscintigraphy in sentinel lymph node detection in patients with melanoma: a meta-analysis. J Clin Med 9(6):1680 10.3390/jcm9061680 PMC7356992 32498217

[49] Lockau H (2018). Dynamic (18)F-FDG PET Lymphography for in vivo identification of lymph node metastases in murine melanoma. J Nucl Med.

[50] Berzaczy D et al (2020) Whole-Body [(18)F]FDG-PET/MRI vs. [(18)F]FDG-PET/CT in Malignant Melanoma. Mol Imaging Biol 22(3):739–744 10.1007/s11307-019-01413-7 31363965

[51] Vianello F (2006). Murine B16 melanomas expressing high levels of the chemokine stromal-derived factor-1/CXCL12 induce tumor-specific T cell chemorepulsion and escape from immune control. J Immunol.

[52] Gretz JE (2000). Lymph-borne chemokines and other low molecular weight molecules reach high endothelial venules via specialized conduits while a functional barrier limits access to the lymphocyte microenvironments in lymph node cortex. J Exp Med.

[53] Li Z et al (2016) Influence of molecular size on tissue distribution of antibody fragments. mAbs 8(1):113–119 10.1080/19420862.2015.1111497 PMC5040103 26496429

[54] Krishnan G (2021). Metastatic and sentinel lymph node mapping using intravenously delivered Panitumumab-IRDye800CW. Theranostics.

[55] Lugade AA (2005). Local radiation therapy of b16 melanoma tumors increases the generation of tumor antigen-specific effector cells that traffic to the tumor. J Immunol.

[56] Hackethal A (2018). Role of indocyanine green in fluorescence imaging with near-infrared light to identify sentinel lymph nodes, lymphatic vessels and pathways prior to surgery - a critical evaluation of options. Geburtshilfe Frauenheilkd.

[57] Knackstedt R (2019). Indocyanine Green fluorescence imaging with lymphoscintigraphy for sentinel node biopsy in melanoma: increasing the sentinel lymph node-positive rate. Ann Surg Oncol.

[58] Kwon S, Sevick-Muraca EM (2007). Non-invasive quantitative imaging of lymph function in mice. Lymphat Res Biol.

[59] Sharma R (2007). Quantitative imaging of lymph function. Am J Physiol Heart Circ Physiol.

[60] Escuin-Ordinas H (2013). PET imaging to non-invasively study immune activation leading to antitumor responses with a 4–1BB agonistic antibody. J Immunother Cancer.

[61] Deichen JT (2003). Uptake of [18F]fluorodeoxyglucose in human monocyte-macrophages in vitro. Eur J Nucl Med Mol Imaging.

[62] Kim EJ (2014). Metabolic activity of the spleen and bone marrow in patients with acute myocardial infarction evaluated by 18f-fluorodeoxyglucose positron emission tomograpic imaging. Circ Cardiovasc Imaging.

[63] Singh B (2008). Preoperative 18F-FDG-PET/CT imaging and sentinel node biopsy in the detection of regional lymph node metastases in malignant melanoma. Melanoma Res.

[64] Crippa F (2000). Which kinds of lymph node metastases can FDG PET detect? A clinical study in melanoma. J Nucl Med.

[65] Vidya R (2019). Diagnostic application of patent blue V in sentinel lymph node biopsy for breast cancer - Is it time for a change?. Indian J Cancer.

[66] Bae MR (2020). (18)F-FDG PET/CT versus CT/MR imaging for detection of neck lymph node metastasis in palpably node-negative oral cavity cancer. J Cancer Res Clin Oncol.

[67] Tanaka Y (2019). Sentinel lymph node-targeted therapy by oncolytic sendai virus suppresses micrometastasis of head and neck squamous cell carcinoma in an orthotopic nude mouse model. Mol Cancer Ther.

[68] Traenkle B et al (2021) Single-domain antibodies for targeting, detection, and in vivo imaging of human CD4+ cells. Front Immunol 12:799910 10.3389/fimmu.2021.799910 PMC8696186 34956237

[69] Almuhaideb A, Papathanasiou N, Bomanji J (2011). 18F-FDG PET/CT imaging in oncology. Ann Saudi Med.

[70] Weiler M, Dixon JB (2013). Differential transport function of lymphatic vessels in the rat tail model and the long-term effects of Indocyanine Green as assessed with near-infrared imaging. Front Physiol.

[71] Carr JA (2018). Shortwave infrared fluorescence imaging with the clinically approved near-infrared dye indocyanine green. Proc Natl Acad Sci.

[72] Griessinger CM (2020). The PET-Tracer (89)Zr-Df-IAB22M2C enables monitoring of intratumoral CD8 T-cell infiltrates in tumor-bearing humanized mice after t-cell bispecific antibody treatment. Cancer Res.

