# Supplementary material for: Preclinical Identification Of Tumor-Draining Lymph Nodes Using a Multimodal Non-invasive In vivo Imaging Approach
Source: Mol Imaging Biol. 2023 Jan 4;25(3):606–18. doi: 10.1007/s11307-022-01797-z (PMC10172276; doi:10.1007/s11307-022-01797-z)
Supplement: Supplementary file 1 — Supplementary file1 (DOCX 410 KB) [file 11307_2022_1797_MOESM1_ESM.docx]

**SUPPLEMENTAL FIGURE S1**

SUPPLEMENTAL FIGURE S1: Measurements of Patent Blue V phantoms for determination of filter settings in the *in vivo* OI analysis. Representative illustrations of the excitation (570 nm, 605 nm, 640 nm, 675 nm, 710 nm) and emission (620 nm, 640 nm, 680 nm, 700 nm, 720 nm, 740 nm, 760 nm, 780 nm) filters that were examined with the respective A: exposure times as well as B: illustrations of the 15 ml phantoms with 4 µmol l^-1^ Patent V Blue in PBS (left phantom) or PBS with 33 g l^-1^ BSA (right phantom) were imaged with the IVIS® system (medium binning, FOV: 25, F-stop: 2).
